# 超临界流体色谱固定相的发展及在天然产物中的应用

**DOI:** 10.3724/SP.J.1123.2023.07024

**Published:** 2023-10-08

**Authors:** Chunying SONG, Gaowa JIN, Dongping YU, Donghai XIA, Jing FENG, Zhimou GUO, Xinmiao LIANG

**Affiliations:** 1.中国科学院大连化学物理研究所, 中国科学院分离分析化学重点实验室, 辽宁 大连 116023; 1. CAS Key Laboratory of Separation Science for Analytical Chemistry, Dalian Institute of Chemical Physics, Chinese Academy of Sciences, Dalian 116023, China; 2.中国科学院大学, 北京 100049; 2. University of Chinese Academy of Sciences, Beijing 100049, China; 3.赣江中药创新中心, 江西 南昌 330000; 3. Ganjiang Chinese Medicine Innovation Center, Nanchang 330000, China

**Keywords:** 超临界流体色谱, 非手性固定相, 天然产物的分离, 综述, supercritical fluid chromatography (SFC), achiral stationary phase, separation of natural products, review

## Abstract

超临界流体色谱(SFC)是以超临界流体为流动相的一种色谱方法。最广泛使用的流动相为CO_2_,可以与多种极性有机溶剂混匀,这种广泛的混溶性使得SFC流动相的极性能够扩展至比正相色谱(NPLC)和反相色谱(RPLC)流动相更宽的范围。流动相兼容的特点决定了固定相的多样性,几乎液相色谱所有的固定相都可以应用在SFC上,既包括RPLC常用到的C18等非极性固定相,也包括NPLC常用到的硅胶等极性固定相。分析物的选择范围也得到了有效扩展,从脂类化合物逐渐发展到黄酮、皂苷及多肽等极性化合物。在SFC中,分析物的分离效果更依赖于固定相的选择。在沿用HPLC固定相的基础上,研究者也在不断地开发更适合SFC的专属固定相。多种多样的固定相共同推进了SFC在多个领域的应用,如制药、食品、环境以及天然产物等。其中天然产物因成分复杂且大多成分含量甚微而成为最有挑战的分离对象之一。得益于仪器的进步以及相关理论体系的健全,SFC的优势逐渐显现,利用SFC分离天然产物的应用也在日益增多。在过去的50年里,SFC已经发展成一种被广泛使用的高效分离技术。本文先简单地描述了SFC的特点优势以及发展过程,然后对近10年来SFC固定相的种类以及在天然产物上的应用进行了综述,并对SFC未来的发展做出了展望。

## Abstract (en)

Supercritical fluid chromatography (SFC) is an environment-friendly and efficient column chromatography technology that was developed to expand the application range of high performance liquid chromatography (HPLC) using a supercritical fluid as the mobile phase. A supercritical fluid has a temperature and pressure that are above the critical values as well as relatively dynamic characteristics that are between those of a gas and liquid. Supercritical fluids combine the advantages of high solubility and diffusion, as their diffusion and viscosity coefficients are equivalent to those of a gas, while maintaining a density that is comparable with that of a liquid. Owing to the remarkable compressibility of supercritical fluids, analyte retention in SFC is significantly influenced by the density of the mobile phase. Thus, the column temperature and back pressure are crucial variables that regulate analyte retention in SFC. Increasing the back pressure can increase the density and solubility of the mobile phase, leading to reductions in retention time. The column temperature can affect selectivity and retention, and the degree to which different analytes are affected by this property varies. On the one hand, increasing the temperature reduces the density of the mobile phase, thereby extending the retention time of the analytes; on the other hand, it can also increase the energy of molecules, leading to a shorter retention time of the analyte on the stationary phase. CO_2_, the most widely employed supercritical fluid to date, presents moderate critical conditions and, more importantly, is miscible with a variety of polar organic solvents, including small quantities of water. In comparison with the mobile phases used in normal-phase liquid chromatography (NPLC) and reversed-phase liquid chromatography (RPLC), the mobile phase for SFC has a polarity that can be extended over a wide range on account of its extensive miscibility. The compatibility of the mobile phase determines the diversity of the stationary phase. Nearly all stationary phases for HPLC, including the nonpolar stationary phases commonly used for RPLC and the polar stationary phases commonly used for NPLC, can be applied to SFC. Because all stationary phases can use the same mobile-phase composition, chromatographic columns with completely different polarities can be employed in SFC. The selectivity of SFC has been effectively expanded, and the technique can be used for the separation of diverse analytes ranging from lipid compounds to polar compounds such as flavonoids, saponins, and peptides. The choice of stationary phase has a great impact on the separation effect of analytes in SFC. As new stationary phases for HPLC are constantly investigated, specialized stationary phases for SFC have also been continuously developed. Researchers have discovered that polar stationary phases containing nitrogen heterocycles such as 2-EP and PIC are highly suitable for SFC because they can effectively manage the peak shape of alkaline compounds and provide good selectivity in separating acidic and neutral compounds.The development of various stationary phases has promoted the applications of SFC in numerous fields such as pharmaceuticals, food production, environmental protection, and natural products. In particular, natural products have specific active skeletons, multiple active groups, and excellent biological activity; hence, these materials can provide many new opportunities for the discovery of novel drugs. According to reports, compounds related to natural products account for 80% of all commercial drugs. However, natural products are among the most challenging compounds to separate because of their complex composition and low concentration of active ingredients. Thus, superior chromatographic methods are required to enable the qualitative and quantitative analysis of natural products. Thanks to technological improvements and a good theoretical framework, the benefits of SFC are gradually becoming more apparent, and its use in separating natural products is expanding. Indeed, in the past 50 years, SFC has developed into a widely used and efficient separation technology. This article provides a brief overview of the characteristics, advantages, and development process of SFC; reviews the available SFC stationary phases and their applications in natural products over the last decade; and discusses prospects on the future development of SFC.

超临界流体色谱(supercritical fluid chromatography, SFC)是为了扩宽高效液相色谱(HPLC)的应用范围而发展起来的一种绿色环保高效的柱色谱技术^[1,2]^。CO_2_是最常用流动相,可以与不同极性的有机溶剂混匀^[3]^。流动相兼容的特点决定了固定相种类的丰富性,几乎液相色谱上所有的固定相都可以应用在SFC上,有效拓宽了分析物的极性范围^[4⇓-6]^,使得SFC在制药、食品、环境以及天然产物等多个领域发挥重要作用^[7]^。其中天然产物的分离分析是科研领域有挑战的方向之一,因为成分复杂且大多数含量甚微,需要更加优异的色谱技术来解决分离和定量困难的问题。得益于仪器的进步,SFC的优势逐渐显现^[8,9]^,利用SFC分离天然产物的趋势也在日益增加^[10,11]^。本文从超临界流体的特殊性质及SFC的发展过程等方面介绍了SFC的研究进展,并综述了SFC固定相在过去10年的发展以及在天然产物中的应用,以期增加科研人员对SFC的了解,促进SFC的发展。

## 1 超临界流体色谱

超临界流体色谱是使用超过临界温度和临界压力的流体作为流动相进行分析的一种色谱技术。SFC采用超临界流体作为流动相,相对动力学性质介于气体和液体之间。扩散系数和黏度系数与气体近似,密度与液体近似,因此SFC综合了高溶解性和高扩散性的优势^[2]^。如图1所示,从分离模式的角度分析,SFC与HPLC非常类似,流动相都发挥着重要作用,不仅体现在分析物会直接溶解于流动相中,还体现在流动相会与分析物竞争固定相表面,从而影响分析物与固定相之间的相互作用^[12]^。但同HPLC所用的液体流动相相比,超临界流体的黏度低,扩散快,表面张力小,样品在SFC上的分离速度更快^[13]^。超临界流体是高度可压缩的,流动相密度对分析物保留的影响很大。因此在SFC中,柱温和背压是调整分析物保留的重要参数^[14]^。增加背压,流动相的密度会变大,溶解能力增强,保留时间缩短。柱温会带来双重的影响,一方面升高温度会降低流动相的密度,削弱溶解能力,保留时间延长;另一方面升高温度可增加分析物分子的能量,保留时间缩短^[15]^。尽管可以通过调节溶剂密度来改变化合物的保留,但是并不能充分改变溶剂的极性。目前使用最多的超临界流体是CO_2_,从图2的相图可以看出,它的临界条件较温和(临界温度*T*_c_=31 ℃,临界压力*P*_c_=7.4 MPa),更重要的是CO_2_具有很好的互溶性,能够与强极性有机溶剂(甚至是微量水)混溶^[16]^。这样的性质使得SFC流动相的极性范围得到了真正的改善,能够扩展至比正相色谱(NPLC)和反相色谱(RPLC)更宽的范围。除了上述独特的性质外,基于CO_2_的流动相还可以兼容多种固定相。在HPLC中,根据色谱模式的不同,固定相的划分比较明确,如硅胶柱只适用于NPLC,而C18色谱柱只能应用于RPLC。但是在SFC中,色谱柱的极性范围可以从C18色谱柱逐渐过渡到硅胶柱。另外得益于商品化SFC仪器的发展,其稳定性、重复性和精密性显著提升,SFC逐渐变成主流的柱色谱技术之一,在多种分离场景下表现出超越HPLC的分离能力^[17]^。

**图 1:**
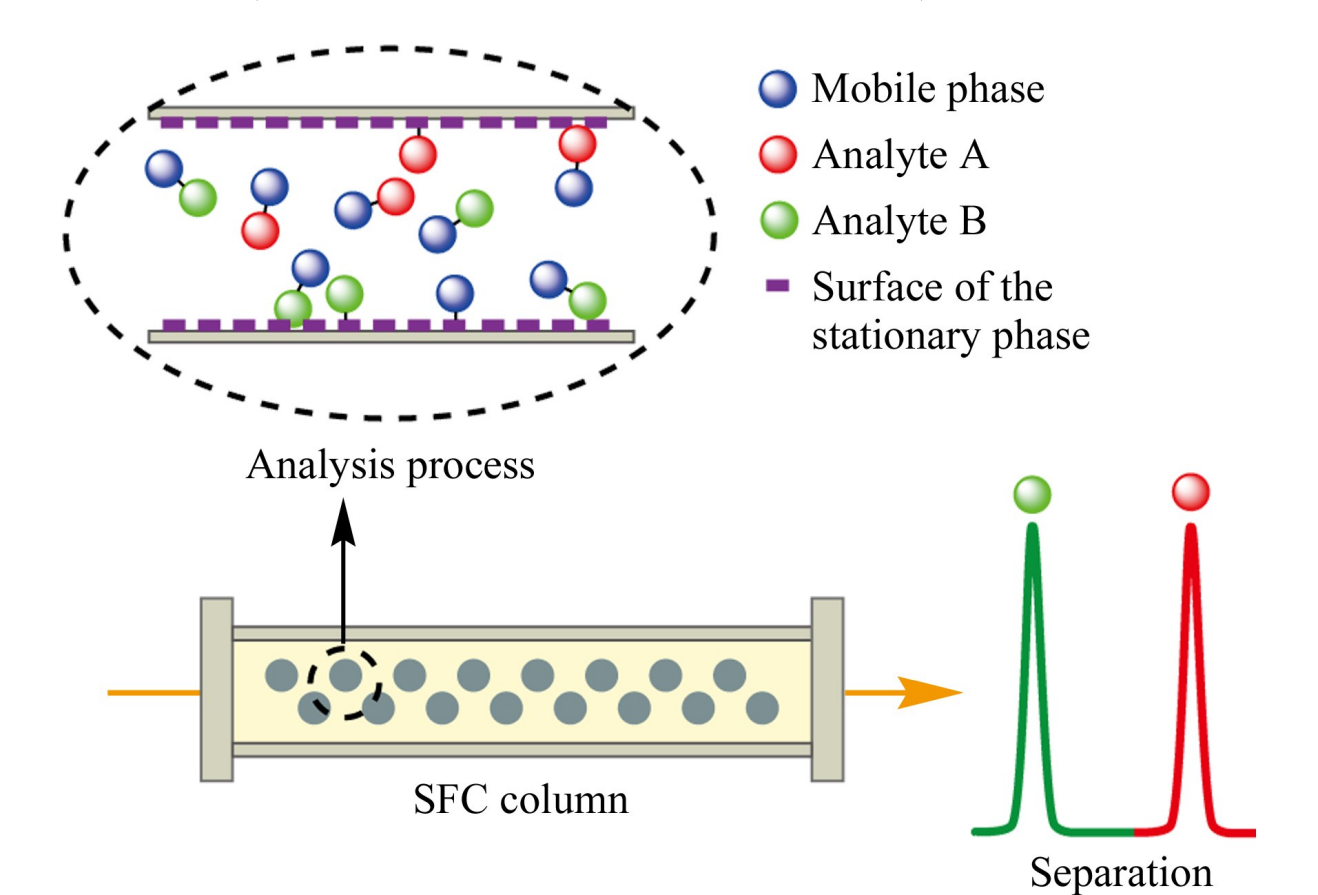
SFC的分离原理

**图 2:**
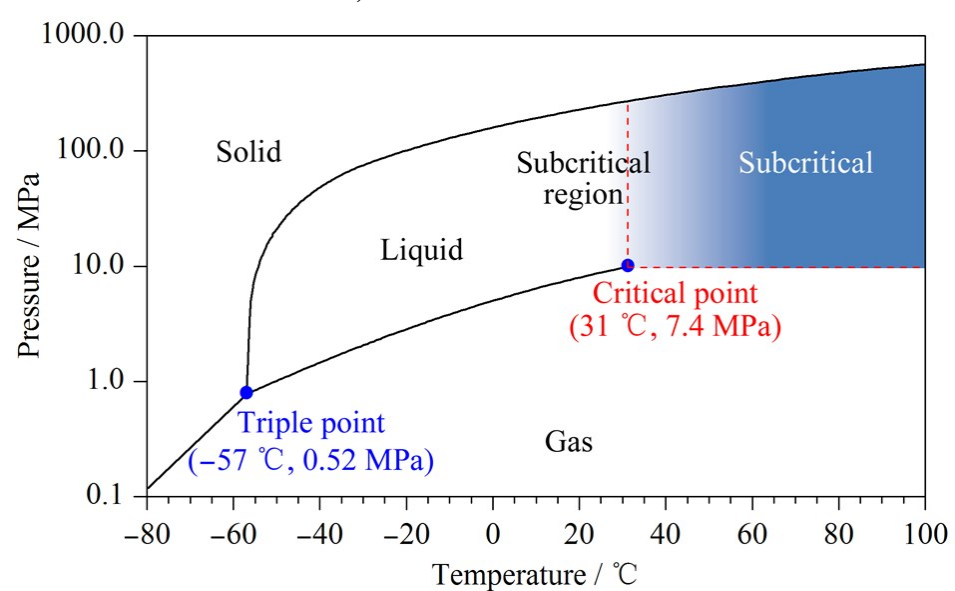
二氧化碳的相图

应用SFC分离样品时,首先要了解分析物的性质,一般认为可以溶于常见有机溶剂的化合物就可以通过SFC分析。为了科学严谨地表明能应用于SFC分析物的适用范围,Berger^[18]^汇总了不同类型的化合物在SFC上的保留,发现弱极性到中等极性的化合物更适合SFC的分析。根据分析物的极性以及分离要求,挑选出最有潜力的固定相进行后续实验。为了得到合适的保留时间和理想的选择性,一般需要加入助溶剂以及添加剂。常用的助溶剂包括甲醇、乙醇、异丙醇以及乙腈等,为了提高选择性,也会选择两种或多种常见的助溶剂混合使用。分析物的酸碱性会影响色谱峰的形状。一般来说,当分析物为酸性时,会选择甲酸、三氟乙酸等作为添加剂^[19]^;当分析物为碱性时,会选择添加三乙胺、氨水等来改善峰形^[20]^。有时为了增加流动相的极性以及改善分析物的峰形,会选择少量水作为添加剂^[21]^。在实验操作中,也会通过调节背压、柱温等参数来获得更好的分离效果。

## 2 SFC固定相的发展

SFC应用初期,毛细管柱是主要的固定相,对很多物质的保留都有限制。而后随着仪器的发展,SFC固定相逐渐从毛细管柱过渡到常规填充柱,体现出应用于SFC的分析物的极性范围也在不断扩大。但是早期并没有专门为SFC研发的固定相,而是延续使用HPLC的固定相^[22]^。

了解分析物的性质与色谱柱表面基团之间的相互作用对于选择最合适的固定相很有必要。选择极性柱,如硅胶柱、氰基、氨基和丙二醇键合相时,SFC以正相模式进行分离,应用对象以极性化合物为主(生物碱、黄酮等)。而当选择非极性和疏水性色谱柱,如C8、C18、C30柱以及苯基柱,SFC则以反相模式运行,应用对象以非极性化合物为主(脂肪酸、脂溶性维生素等)^[23]^。

当SFC的发展越来越成熟后,科研人员不再满足于继续沿用HPLC的色谱柱。2001年,2-乙基吡啶(2-EP)固定相的出现为发展适合SFC的固定相提供了新的思路。2-EP的保留机理主要有3个:吡啶基团与固定相上的硅醇基的氢键作用使固定相活性降低;质子化后的吡啶与分析物中带正电的基团之间的电荷排斥作用;中性吡啶基对硅胶固定相活性位点的立体屏蔽效应^[24]^。在使用过程中发现,同其他色谱柱相比,2-EP对碱性化合物的拖尾有很好的抑制效果,同时在分离酸性和中性化合物时也可以提供良好的选择性^[25]^。随后,Waters公司相继推出了多款性能更优异的SFC键合相,包括PIC、DIOL、DEA和1-AA等,满足了更多分离体系的需求。Shimadzu公司也合成了14款专用于SFC的固定相,Gros等^[26]^利用线性溶剂化能量关系模型研究了以上色谱柱的保留机理,以方便使用者根据分析物的特点选择合适的固定相类型。与此同时,科研人员也在推出专门为SFC发展的键合相。如McClain等^[27]^设计并合成了一系列含有氢键基团的杂环固定相,这些固定相很容易与分析物形成多点相互作用,其中2-PY-U和4-PY-A显示出与商品化固定相相当的色谱性能和通用性。更重要的是,McClain等^[27]^考虑了固定相结构对色谱性能和通用性的影响,提出了SFC固定相的设计原则:在保留含氮杂环的基础上,增加氢键等多种相互作用,以利于与分析物形成多个吸附位点。该观点为后续SFC固定相的设计提出了思路和方向。Bocian等^[28]^合成了一系列含有苯基的固定相并进行了吸附测试。观察到有机溶剂的吸附在很大程度上取决于键合相中极性官能团的存在,这些极性官能团影响固定相表面的疏水性和极性。Borges-Munoz等^[29]^合成了C18酰胺固定相,同硅胶柱或末端酰胺柱相比,该固定相对极性化合物的保留较弱,但却表现出良好的选择性。Ovchinnikov等^[30]^首次将两性离子亲水固定相应用在SFC上,并利用线性溶剂化能量关系模型对该色谱柱对化合物的保留进行分析,发现主要作用力来自氢键相互作用,保留特性与2-EP接近。Nagai等^[31]^设计合成了一种聚对苯二甲酸丁二醇酯的新型固定相,该固定相由平面芳香苯基和酯基单体单元组成,显示出对同分异构芳烃和类似化合物的优异识别能力。West等^[32]^设计合成了3种基于二氧化硅的聚乙烯吡啶固定相,与2-EP和2-PIC相比具有更强的保留和更好的屏蔽残留硅醇基的作用。Fu等^[33]^合成并评价了一系列键合密度不同的C8固定相,以扩大SFC的应用范围。同硅胶柱相比,该固定相虽然保留较弱,但选择性更加优异,可以区分不同烷基链、双键和顺反式结构的生物碱,进一步证明了烷基固定相在提高SFC选择性方面的潜力。Jiang等^[34]^合成了一系列具有不同取代基的苯基型固定相,利用线性溶剂化能量关系模型研究了这些固定相在SFC中的保留机制,并利用25种酚类化合物研究色谱柱之间良好的正交性。Wolrab等^[35]^合成了6种混合模式的离子交换色谱柱,研究了碱性和两性离子分析物在HPLC和SFC模式下的分离性能。结果显示,不同色谱模式下,离子交换作用都是主要作用力,并对碱性化合物表现出很好的分离效果。Fu等^[36]^在SFC中首次使用反相色谱/离子色谱(RP/IC)混合模式固定相,并将其应用于吲哚类生物碱的快速分离中。综合来看,SFC上键合相的发展方向是多元化的,不局限于某种类型(以上固定相的汇总见表1)。

**表 1:** SFC固定相的汇总

Column name		Bonded phase structure			Material property					Source	
Diol		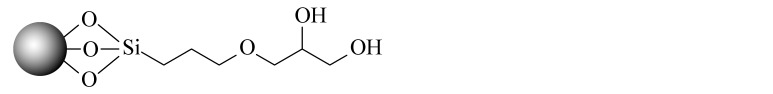			commercially available					Waters	
1-AA		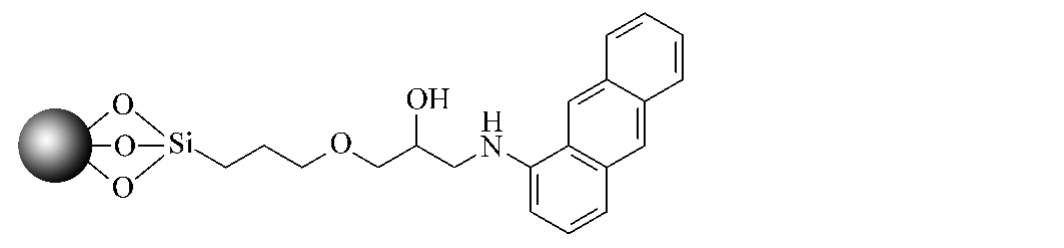			commercially available					Waters	
2-PIC		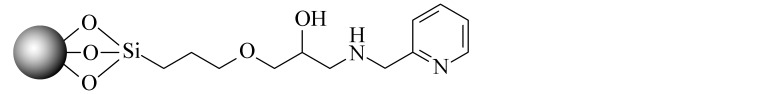			commercially available					Waters	
DEA		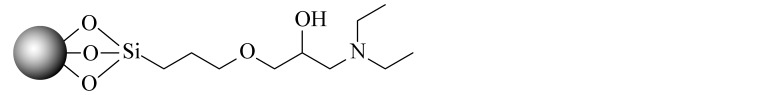			commercially available					Waters	
NH2		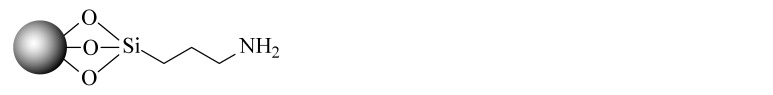			commercially available					Shimadzu	
CN		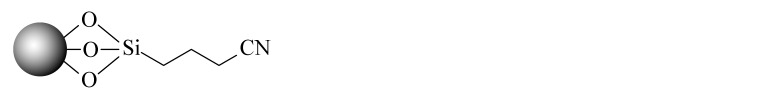			commercially available					Shimadzu	
Amide		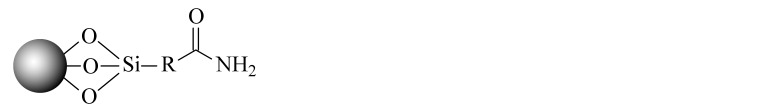			commercially available					Shimadzu	
Diol		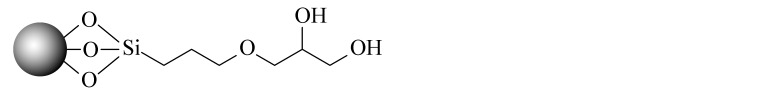			commercially available					Shimadzu	
Sil		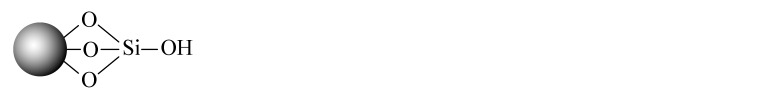			commercially available					Shimadzu	
Triazole		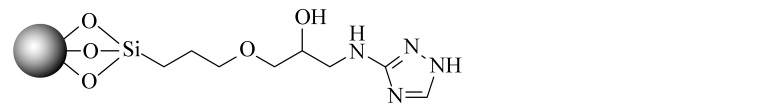			commercially available					Shimadzu	
Py		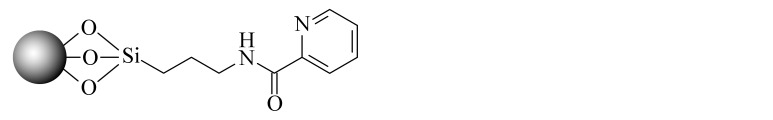			commercially available					Shimadzu	
HyP		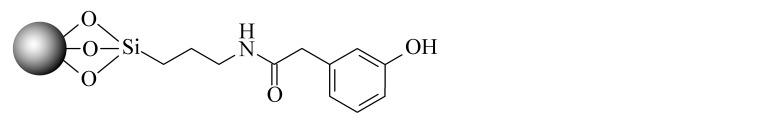			commercially available					Shimadzu	
RP		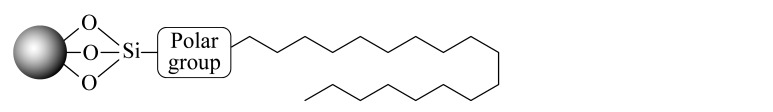			commercially available					Shimadzu	
GIS Ⅱ		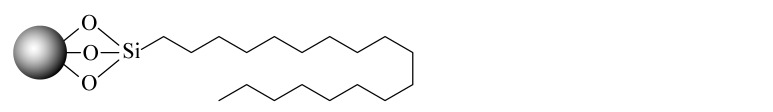			commercially available					Shimadzu	
PBr		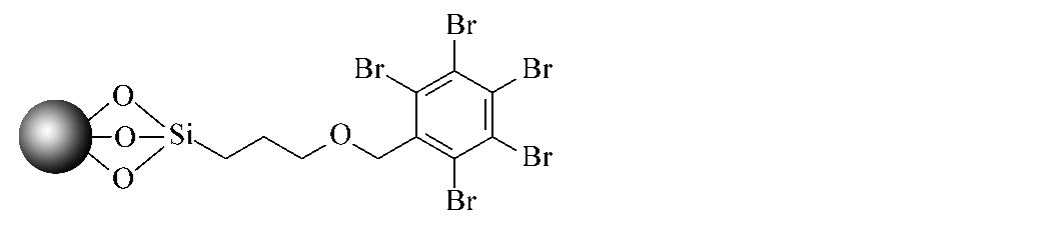			commercially available					Shimadzu	
Choles		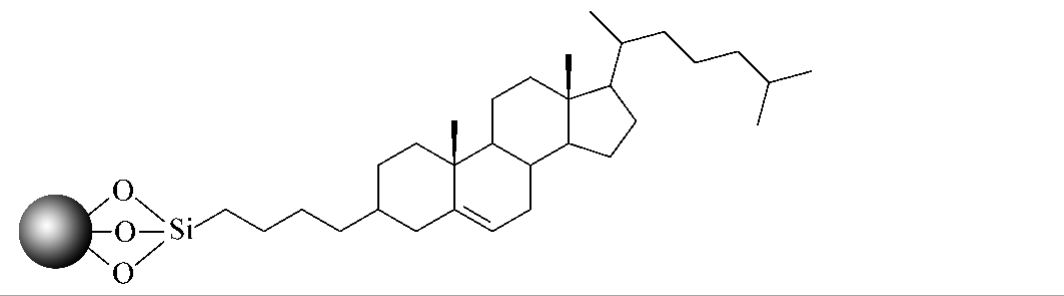			commercially available					Shimadzu	
Phenyl		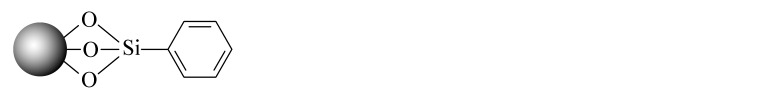		commercially available				Shimadzu			
PyE		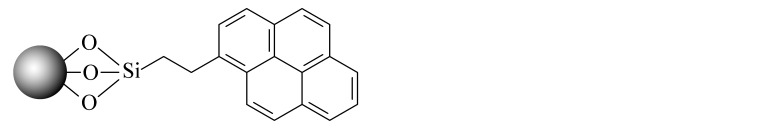		commercially available				Shimadzu			
2-EP		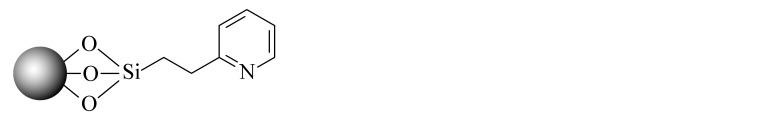		commercially available				Princeton			
PPA		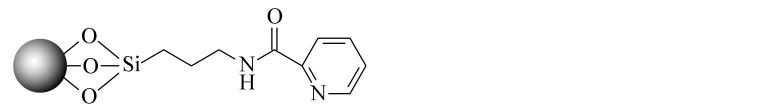		commercially available				Princeton			
PPU		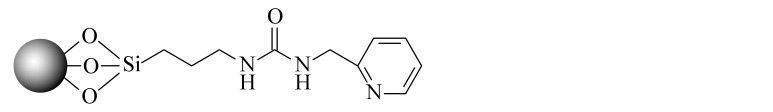		commercially available				Princeton			
2-Py-A		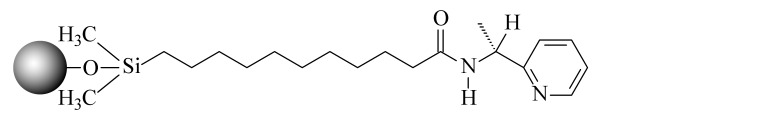		scientific research				[27]			
3-Py-A		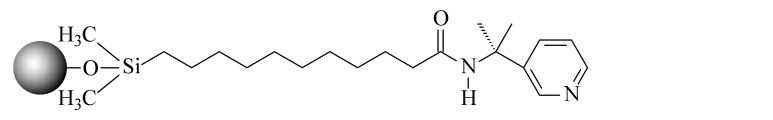		scientific research				[27]			
4-Py-A		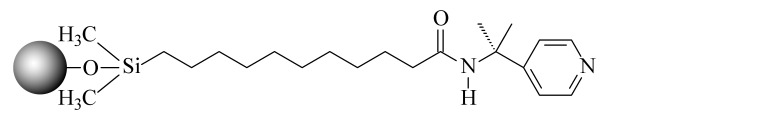		scientific research				[27]			
Pz-A		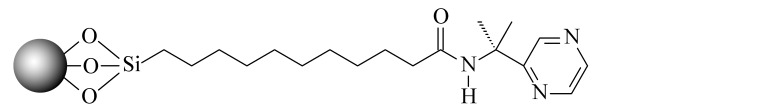		scientific research				[27]			
Q-A		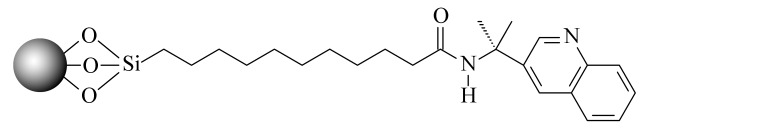		scientific research				[27]			
Q-S		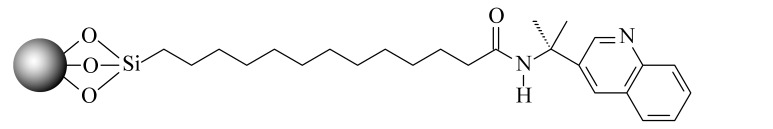		scientific research				[27]			
2-Py-U		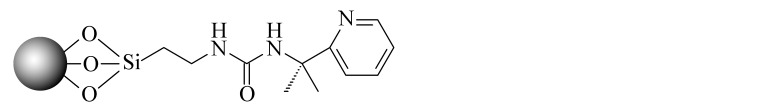		scientific research				[27]			
3-Py-U		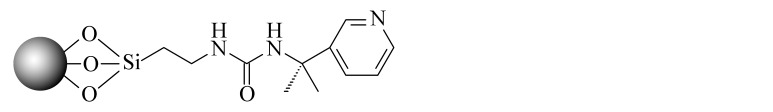		scientific research				[27]			
4-Py-U		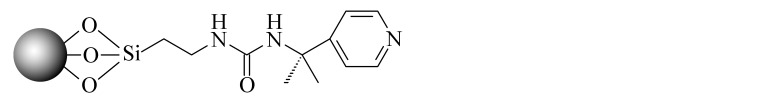		scientific research				[27]			
Pz-U		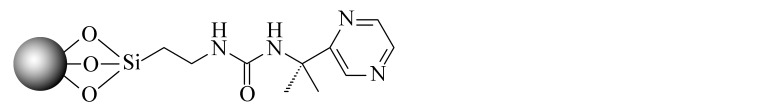		scientific research				[27]			
Q-U		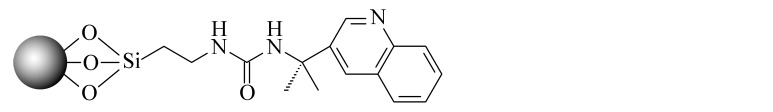		scientific research				[27]			
Phenyl-propyl		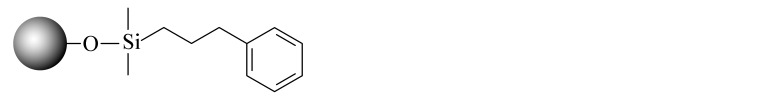			scientific research			[28]		
Phenoxy-propyl		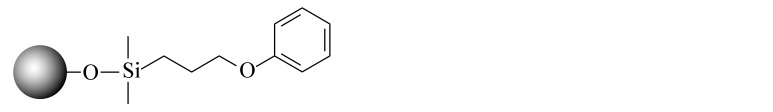			scientific research			[28]		
Phenyl-amide		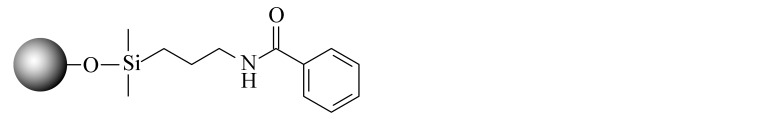			scientific research			[28]		
Phenyl-amine		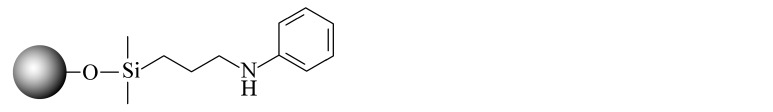			scientific research			[28]		
Phenyl-hexyl		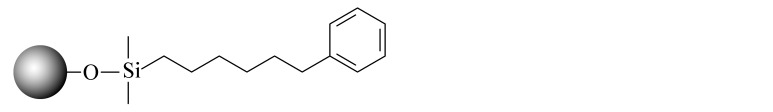			scientific research			[28]		
Phenyl hydride		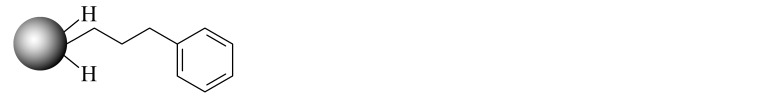			scientific research			[28]		
C18-amide		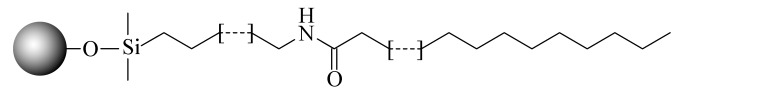			commercially available			MAC-MOD		
Nucleodur HILIC		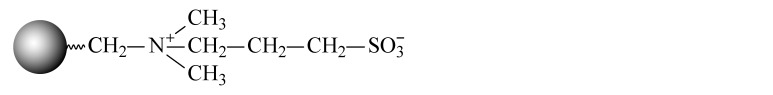			commercially available			Macherey- Nagel		
PBT		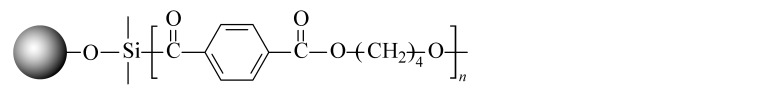			scientific research			[31]		
P2VP		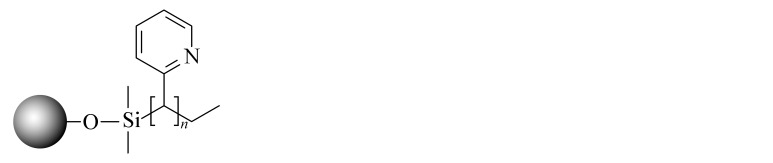			scientific research			[32]		
P3VP		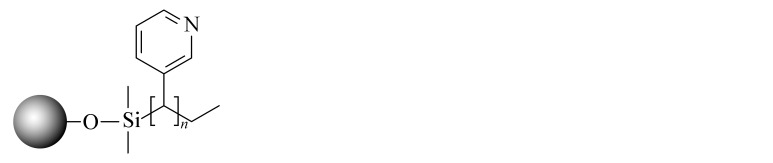			scientific research			[32]		
P4VP		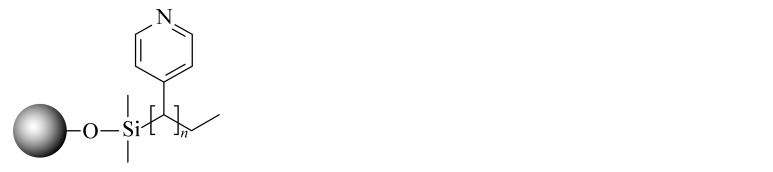			scientific research			[32]		
C8		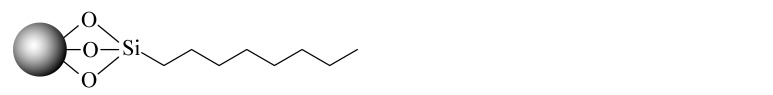			scientific research			[33]		
PB		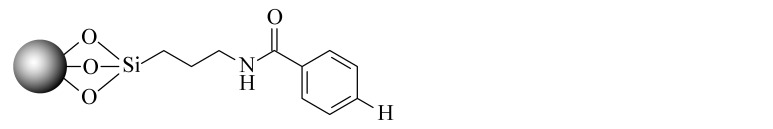			scientific research			[34]		
PB-F		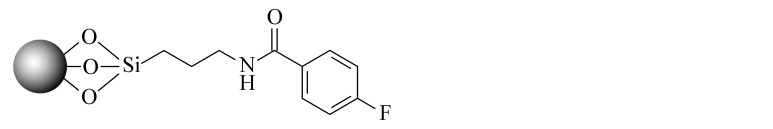			scientific research			[34]		
PB-ET		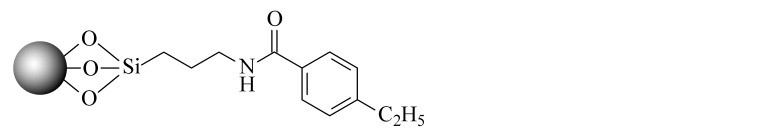			scientific research			[34]		
ZEX1	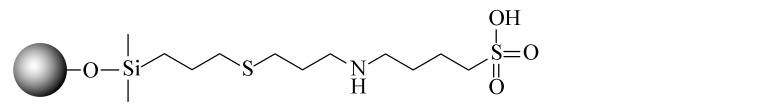			scientific research				[35]
ZEX2	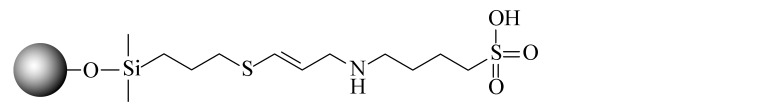			scientific research				[35]
ZEX3	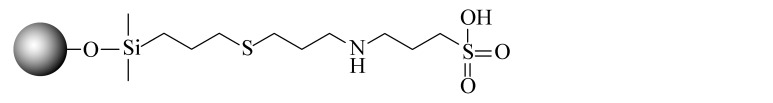			scientific research				[35]
SCX1	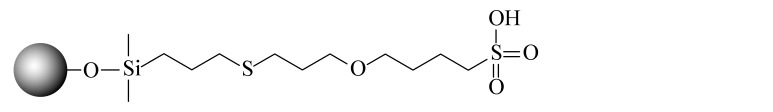			scientific research				[35]
SCX2	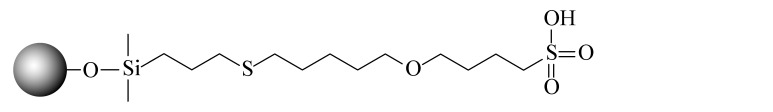			scientific research				[35]
SCX3	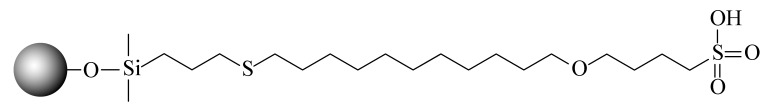			scientific research				[35]
C8SAX	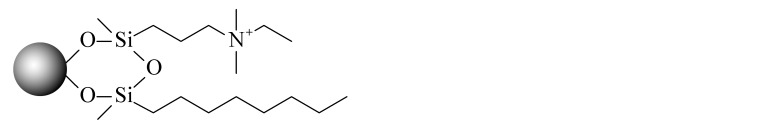			scientific research				[36]
C8SCX	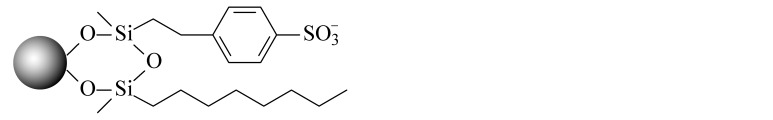			scientific research				[36]

在键合相种类不断发展的过程中,SFC色谱柱的粒径也在逐渐向更小的方向发展。Perrenoud等^[37]^在UHPLC和UHPSFC上分别对比了粒径为1.7 μm和3.5 μm的完全多孔填充色谱柱的动力学性能以及归一化的压降随线速度的变化,结果表明两个规格的色谱柱在UHPSFC上的动力学性能明显优于UHPLC,但是两根色谱柱在UHPSFC上的压降却低于UHPLC,体现了超临界流体黏度低、扩散快的优势。另外在UHPSFC上,1.7 μm的色谱柱最优线速度区间更大,即亚2 μm小粒径色谱柱在高流速下依然保持高效分离的能力。同时为了将柱外效应降到最低,一般会将色谱柱的规格做到100 mm×3 mm或更大规格^[38]^。目前亚2 μm的材料可以与现有SFC仪器完美匹配,应用的场景也越来越多^[39]^。Khalikova等^[40]^利用1.7 μm的PIC色谱柱建立了快速的SFC方法来测定9种防晒剂的含量,并对一款市售防晒霜的成分进行了测定。Novakova等^[41]^开发了一种用于9种雌激素衍生物的超快速定量方法。对固定相种类、改性剂种类、柱温、背压和梯度条件等色谱参数进行了单变量的筛选,最终利用1.7 μm的PFP色谱柱在1.6 min内完成对上述化合物的基线分离。Du等^[42]^利用1.7 μm的BEH色谱柱建立了10种生物胺的SFC分离方法,并将该方法应用于鱼和虾体内生物胺的检测。

## 3 SFC对天然产物的分离

天然产物具有特定的活性骨架和活性基团以及优异的生物活性,为新药发现提供了许多新机会。据报道,与天然产物有关的化合物在商品药中占80%^[43]^。植物是天然产物的重要来源,含有黄酮类、生物碱类、萜类等成分,这些成分是中药材发挥药效作用的重要物质基础。SFC具备中药材分离和纯化方面的优势,几类中药材中重要的活性物质在SFC上的分离情况总结如下。

### 3.1 脂类

脂类是中药中主要的一类化学成分,疏水酰基链和不同类型的亲水基团之间复杂的组合方式使得脂类的种类丰富,包括脂肪酸、酰基甘油、磷脂等。脂类是SFC发展初期主要的应用对象,也是目前为止在SFC上研究最多的一类化合物^[44]^。早期的研究中一般使用超临界毛细管柱分析极性较弱的脂类。如Baiocchi等^[45]^在考察了多个色谱条件对分离的影响以及对比了几种不同极性的固定相后,建立了在5 min内快速分离脂类的方法,并从植物油和鱼油中分离了13种三酰甘油。BorchJensen等^[46]^建立了一种利用DB-225极性毛细管柱分离蓖麻籽油和雨环菊种子油中几种游离脂肪酸的方法,并经过质谱鉴定,纯度为87%。随着超临界柱色谱技术的发展,SFC上可以分离的脂质的极性范围逐渐扩大。常用的色谱柱包括氰丙基柱、氨基柱、氰基柱、硅胶柱、C8柱和C18柱^[47⇓-49]^。探索脂类的分离规律时,研究人员发现了C18等反相柱在SFC模式下优异的分离效果,提高了对SFC分离机理的认知水平,并扩大了SFC色谱柱的范围。如Matsubara等^[50]^比较了类胡萝卜素及其环氧化产物在硅胶柱、苯基柱、C18柱和C30柱上的分离效果,发现在C18柱上得到了最佳的分离度和最短的分析时间。Choo等^[51]^利用C18柱来分离棕榈油中的脂肪酸、生育酚、甘油三酯以及胡萝卜素等成分。Tyskiewicz等^[52]^利用C18柱对废鱼油中的脂溶性维生素进行了分离。虽然固定相是决定化合物选择性的主要因素,但是优化改性剂的种类也是有必要的。最新的报道中,Kozlov等^[53]^对甘油异构体分离时发现了不同溶剂对选择性和峰形的影响,当改性剂为甲醇时,分离性能最优。

### 3.2 萜类

萜类及其衍生物是由戊二羟酸构成的具有异戊二烯结构特征的一类化合物^[54]^。在自然界中广泛存在,具有抗氧化和抗菌的生物活性,以及抗炎和抗癌作用。萜类按其分子中异戊二烯的数量划分,可分为单帖、倍半萜、二萜、三萜和四萜等。然而,各种萜烯的结构密切相关,导致它们的分离变得很复杂。目前主流的分离方法以气相色谱为主,但是这种方法只限于分析水平,不适合大规模制备^[55]^。而SFC有较好的对映体分离能力,同时又可以满足制备需求,正在逐渐成为分离萜类化合物的新主流色谱技术。常用来分析萜类的固定相包括C18柱、苯基己基柱、PFP柱、2-EP柱、硅胶柱和氨基柱等^[56⇓-58]^。一般选择甲醇作为改性剂,很少使用添加剂。Kohler等^[59]^开发了测定青蒿中2种倍半萜(青蒿素和青蒿酸)的超临界柱色谱和超临界毛细管色谱方法。利用氨基柱色谱分离青蒿素和青蒿酸的时间不超过8 min,而超临界毛细管色谱的分离时间约为25 min。与超临界毛细管色谱相比,超临界柱色谱技术具有更高的分析效率、更短的分析时间和更强的分辨能力。Lesellier等^[60]^首次尝试用SFC建立分析三萜类化合物的分离方法,并将该方法应用于苹果渣提取物的分离上,20 min内分离了15种三萜类化合物,显示了该方法在三萜类化合物分离中的巨大潜力。另外利用SFC-MS联用技术对萜类化合物进行高效分离并鉴定也是一个重要的方向。Jones等^[61]^采用SFC-MS对洋甘菊提取物中的倍半萜等成分进行分离和鉴定。同液相色谱方法相比,保留时间更短而且分离效果更好。

### 3.3 生物碱

生物碱是一类含氮的碱性有机化合物,大多数有复杂的氮环结构,具有显著的生物活性,是中草药重要的有效成分之一。分析生物碱常用到的色谱柱有PFP、PIC、2-EP、Torus 1-AA以及Diol柱等,助溶剂的选择主要有甲醇、乙醇和乙腈^[62,63]^。分析物中的氮原子与固定相中残存硅醇基之间的相互作用使得分离生物碱时存在严重的拖尾现象。为了获得正常的峰形,通常选择加入碱性添加剂,如氨水和三乙胺等。Huang等^[64]^在阔叶十大功劳木不同部位测定了8种异喹啉生物碱的含量。经过系统的色谱条件优化,最终确定分析柱为PFP,助溶剂为甲醇,改性剂为氨水和少量的水。上述8种化合物在6 min之内实现了完全分离,同HPLC的分析时间相比缩短了50 min。Yang等^[65]^对钩藤中4种吲哚类生物碱异构体进行了分离和纯化,建立了一个稳定快速的色谱方法,选择1-AA色谱柱,助溶剂为乙腈,添加剂为二乙胺,该方法被证明是快速分析和制备高纯度标准品的可行方案,同时也证明了SFC在对手性化合物分离时的优势。然而分离生物碱时,也存在不加添加剂的情况,此时需要利用固定相表面的静电排斥作用来控制生物碱峰形。如Fu等^[66]^分析雷公藤中的倍半萜吡啶类生物碱时,选择2-EP色谱柱、纯甲醇为改性剂也可以得到比较对称的色谱峰。另外Jiang等^[67]^在分离10种异喹啉生物碱时,首次在SFC中使用低共熔溶剂作为添加剂。同常用的添加剂(甲酸和水)相比,低共熔溶剂可以显著地降低硅醇基的作用,改善生物碱的峰形。多维色谱联用的方式是解决复杂样品体系分离的重要手段,如辛华夏等^[68]^建立了基于反相液相制备色谱和超临界流体制备色谱的组合方法,用于分离纯化醇提水沉后石油醚层中的海风藤。基于两维色谱不同的选择性,从中分离出6种化合物,包括墙草碱等生物碱,显示出超临界流体色谱在天然产物的分析和制备方面的巨大潜力。

### 3.4 黄酮

黄酮类化合物在植物中含量丰富,具有抗氧化和抗炎作用,是药用植物中主要的活性成分^[69]^。分析黄酮常用到的色谱柱包括硅胶柱、Diol柱、苯基柱、C18柱以及PIC柱^[70,71]^。由于酚羟基的存在,黄酮一般在酸性条件下可以获得对称的峰形,常用的酸性添加剂包括甲酸、三氟乙酸以及磷酸。如Huang等^[72]^经过科学的色谱方法优化,在硅胶柱上采用梯度洗脱对12种黄酮类化合物进行分离。其中发现含0.1%磷酸的甲醇溶液是分离黄酮类化合物最合适的极性改性剂。与开发的HPLC相比,分析速度快3倍。当黄酮类化合物中不存在酚羟基时,酸性添加剂的加入不是必要的。Li等^[73]^利用SFC分离橘皮素、褐皮素、橙皮素和柚皮素4种黄酮类化合物时,发现是否在改性剂中加入甲酸,对不同化合物的峰形影响不同。当存在酚羟基时,选择酸性添加剂有利于控制电离状态,从而得到更加对称的峰形。Gibitz-Eisath等^[74]^在分离马鞭草提取物中的8种黄酮类化合物时,对比了HPLC的分离方法,两种色谱模式都能在短时间内达到很好的分离效果,但是化合物的出峰顺序不一样。体现了两种色谱模式高度正交,突出了方法交叉验证在天然产物分析中的重要性,有利于发展多维色谱模式来分离复杂的天然产物。黄酮醇是一类具有药用价值的黄酮类化合物,Liu等^[75]^首次用SFC来分离3种极性黄酮醇异构体,表明超临界流体色谱法在黄酮类化合物的分析、分离制备等方面将有广阔的应用前景。

### 3.5 皂苷

皂苷由一个或多个亲水糖苷部分(葡萄糖、半乳糖、葡萄糖醛酸、木糖等)和一个皂苷元的亲脂苷元连接而成。根据皂苷元的性质,可分为三萜类和甾类。它们通常有重要药理活性,如抗炎、抗过敏、免疫调节和抗病毒等^[76]^。利用SFC分析皂苷的应用相对较少,因为皂苷的极性较强,在SFC上存在保留强洗脱难的问题^[77]^。然而SFC固定相的发展以及多种改性剂的使用,逐渐解决了该问题。迄今为止,多个固定相如2-EP柱、苯基己基柱、氰基柱、Diol柱、C18柱、硅胶柱、PFP柱和苯基柱已被报道用于皂苷的分离。如Sun等^[78]^利用Torus Diol色谱柱建立了分离和定量柴胡皂苷的新方法。通过优化色谱条件,5种柴草皂苷在22 min内成功分离。并将其与HPLC进行比较,发现SFC分离柴胡皂苷时具有分析时间短、分离效果好的显著优势,为皂苷的分离定量提供了一种新的方法。由于皂苷的高极性,一般会在改性剂中加入少量水作为添加剂来提高流动相的洗脱强度。如Huang等^[79]^建立了一种快速高效的SFC-MS联用方法来分离苦藤皂苷、山楂皂苷和人参皂苷。在优化色谱方法的过程中,发现在甲醇中加入少量水(5%~10%)以及甲酸(0.05%)时,化合物的分离程度以及质谱灵敏度都会提高。且利用SFC建立的方法的分析时间是HPLC方法的1/2。超临界流体的密度也可以作为调节流动相洗脱强度的方法。如Xing等^[80]^建立了离线二维SFC-HPLC方法分离三七药材中的三萜皂苷。在优化SFC方法时,选择降低柱温和增加背压的方式来提高洗脱强度,减少分析时间。

利用SFC分离天然产物中活性物质的相关色谱条件汇总见表2。

**表 2:** SFC对天然产物分离的色谱条件汇总

Category	Analytes	Samples		Mobile phase	Stationary phase	Ref.
Lipids	13 triacylglycerols	vegetable and fish oils		CO_2_	OV1	[45]
Lipids	16 free fatty acid deriva- tives	seed oil from ricinus communis and dimorphoteca pluvialis		CO_2_	DB-225	[46]
Lipids	13 carotenoids and epoxy carotenoids	human serum and low-density lipoprotein samples		CO_2_-MeOH (0.1% ammonium formate)	Merck Puroshere RP-18e	[50]
Lipids	fatty acid, tocopherol, tri- acylglycerol, triglyceride, carotene		crude palm oil	CO_2_-EtOH	Super Crestpak C18	[51]
Lipids	14 fat-soluble vitamins		hemp seed oil and waste fish oil	CO_2_-MeOH	Waters HSS C18 SB	[52]
Lipids	28 monoacylglycerol and diacylglycerol isomers		lipases from porcine pancreas and pseudomonas fluorescens	CO_2_-MeOH	Waters Trefoil AMY1 (AMY1)	[53]
Terpenes	artemisinin and artemisin- ic acid		Artemisia annua L.	CO_2_-MeOH	Macherey-Nagel Nucleosil NH_2_	[59]
Terpenes	8 triterpenoid compounds		apple pomace extracts	CO_2_-MeOH	Phenomenex Synergi Polar-RP	[60]
Terpenes	6 sesquiterpene lactones		roman and german chamomile	CO_2_-MeOH/IPA (0.1% FA)	Waters Acquity UPC^2^ BEH 2-EP	[61]
Alkaloids	8 isoquinoline alkaloids		different parts of Mahonia bealei (Fort.) Carr.	CO_2_-MeOH (0.2% ammonia solution, 8% H_2_O)	Dikma Technologies Inspire PFP	[64]
Alkaloids	4 indole alkaloids		no sample	CO_2_-ACN (0.2% DEA)	Waters Torus 1-AA	[65]
Alkaloids	6 sesquiterpene pyridine alkaloids		root bark of T. wilfordii	CO_2_-MeOH	Waters Acquity UPC^2^ BEH 2-EP	[66]
Alkaloids	8 isoquinoline alkaloids		Sinomenii Caulis, Corydalis Rhizoma, Coptidis Rhizoma, Mahoniae Caulis, Phellodendri Chinensis Cortex, and Stephaniae Tetrandrae Radix samples	CO_2_-MeOH (2% H_2_O, 0.5% FA, 0.25% DES)	Agilent Zorbax RX-SIL	[67]
Alkaloids	6 compounds (including alkaloids)		piper kadsura	CO_2_-MeOH	XAmide	[68]
Flavonoids	12 flavonoids		Chrysanthemum morifolium Ramat.	CO_2_-MeOH (0.1%PA)	Agilent Zorbax RX-SIL	[72]
Flavonoids	4 flavonoids		citrus samples	CO_2_-MeOH (1%FA)	Waters Viridis BEH	[73]
Flavonoids	7 flavonoids		Verbena officinalis L. (Verbenaceae) extracts	CO_2_-MeOH (0.15%FA)	Waters Acquity UPC^2^ Torus Diol	[74]
Flavonoids	3 flavonol isomers		no sample.	CO_2_-EtOH (0.5%PA)	phenyl	[75]
Saponins	5 saikosaponins		Chaihu Dropping Pills	CO_2_-MeOH	Waters Acquity UPC^2^ Torus Diol	[78]
Saponins	9 triterpenoid saponins		Ilex latifolia Thunb., Panax quinquefo- lius L. and Panax ginseng C. A. Meyer.	CO_2_-MeOH (10% H_2_O, 0.05% FA)	Agilent Zorbax RX-SIL	[79]
Saponins	triterpenoid saponin		P. notoginseng	CO_2_-MeOH	Waters Atlantis^®^ HILIC Silica	[80]

MeOH: methanol; EtOH: ethanol; IPA: isopropanol; FA: formic acid; ACN: acetonitrile; DEA: diethylaniline; DES: deep eutectic solvents; PA: phosphoric acid.

## 4 总结及展望

经过近50年的不断发展,SFC凭借分离速度快和对异构体选择性好的特点,在手性分离领域快速发展,同时在非手性领域的应用也越来越多。SFC从早期局限于对脂类的分离,逐渐扩展到对黄酮、皂苷甚至多肽等极性化合物的分离。这样的发展与极性助溶剂的加入以及丰富多样固定相的使用息息相关。几乎HPLC上的固定相都可以用在SFC上,范围可以从C18逐渐过渡到硅胶柱。专门为SFC研发的固定相,特别是极性固定相,在分离过程中通过氢键作用、疏水作用以及*π-π*相互作用等多种作用力改善了峰形并提高了选择性。超临界流体的低黏度性,既推动了快速色谱的发展,又促进了小粒径固定相的使用。仪器公司推出了耐高压SFC仪器,与亚2 μm材料完美兼容。同HPLC相比,亚2 μm材料在SFC上的最优线速度更大,理论塔板数最小值更低,展现出优异的动力学性质。随着色谱技术的快速发展,SFC也在复杂体系的分离中逐渐发挥巨大优势。SFC未来的发展兼具创新性和实用性,发展方向有以下几个方面。第一是固定相的发展。包括发展“通用性”SFC固定相,结构较丰富,可能含有杂原子、杂环等结构,保证分离时存在多种相互作用力,适用分析物的极性区间广泛,为大多数化合物和复杂体系提供良好的分离效果;针对难分离的对象,设计合成有特异选择性的固定相,解决这类样品的分离问题;根据超临界流体黏度低、扩散快的特点,发展独特的固定相类型促进传质过程,或许类似于有序晶体这样的结构更适合SFC。第二是耐超高压仪器的研发带动超快速色谱的发展。仪器的耐压程度大幅度提升,将固定相的尺寸带到亚微米时代。从而得到超高柱效,峰容量急速提高,色谱分析速度缩短到以“秒”计时。第三是在非手性应用领域应用的发展。目前最广泛的色谱技术仍然是HPLC,而SFC仅在小分子药物手性分离领域有较大的发展空间。随着认识水平的提高以及仪器的进步,SFC可能会在非手性领域,特别是复杂天然产物的分离中发挥巨大作用。第四是制备型SFC的发展。超临界CO_2_无毒无害,不可燃,而且价格便宜,在制备色谱中有安全经济的巨大优势。但是目前分析和制备过程的转化还存在一定的问题,主要与超临界流体高度可压缩的性质相关。未来还需要解决SFC在分析与制备方法转移之间的制约问题,为纯化制备领域带来效率和经济的提升。
